# Comparison of Differential Flavor Metabolites in Meat of Lubei White Goat, Jining Gray Goat and Boer Goat

**DOI:** 10.3390/metabo9090176

**Published:** 2019-09-05

**Authors:** Weiting Wang, Bei Sun, Peng Hu, Meng Zhou, Sujun Sun, Pengfei Du, Yi Ru, Alexander Suvorov, Yongsheng Li, Yaobo Liu, Shoujing Wang

**Affiliations:** 1 Key Laboratory of Agro-Products Processing Technology of Shandong Province/Key Laboratory of Novel Food Resources Processing, Ministry of Agriculture and Rural Affairs, Institute of Agro-Food Science and Technology, Shandong Academy of Agricultural Sciences, 202 Gongye North Road, Jinan 250100, China (W.W.) (P.H.) (S.S.) (P.F.D.) (Y.R.) (A.S.) (Y.S.L.); 2 Jiangsu Uniwell Biotechnology Co. Ltd., No. 16 Yulan Avenue, Xuyi County Economic Development Zone, Xucheng 211700, China; 3 Jiangsu Provincial Xuzhou Pharmaceutical Vocational college, Xuefu Road, Tongshan District, Xuzhou 221116, China; 4 Jinan Animal Product Quality and Safety Monitoring Center, No. 12 Wanshou Road, Jinan 250100, China

**Keywords:** goat, meat, flavor, metabonomics

## Abstract

Flavor is one of the most important sensory characteristics of meat. The development of taste and aroma can be attributed to thousands of flavor molecules and precursors that are present in meat tissues. As a result, the identification of these flavor compounds and an improved understanding of their roles are necessary for improving the sensory quality and customer appeal of meat products. In the current study, we compared the metabolic profiles of meat specimens from the Lubei white goats (LBB), Boer goats (BE) and Jining grey goats (JNQ) by untargeted liquid chromatography-mass spectrometry. Our metabolomic data revealed that the three types of goat meat showed significantly different profiles of fatty acids, aldehydes, ketones, lactones, alkaloids, flavonoids, phenolics and drug residues, which could underpin the nuances of their flavors. Taken together, our results provided insights into the molecular basis for sensory variations between different goat meat products.

## 1. Introduction

Flavor is a critical sensory attribute of meat and is widely regarded as one of the most important factors related to its quality and customer appeal. Due to the complex chemical composition of meat, its flavor is generally not tied to a single compound, but rather to a wide variety of molecular constituents, particularly carbohydrates, amino acids, carbonyls and lipids [1,2]. For farm animals, compounds derived from feed have also been shown to be able to alter the flavor quality [3]. The various chemical ingredients of raw meat often react with each other during heating, producing new molecules that affect the palatability of the cooked dishes [4]. In particular, the Maillard reaction between carbonyl compounds and amines is responsible for the formation of many meat flavor compounds, such as heterocycles, sulfur-bearing molecules and amino acid-derived aldehydes [5,6]. Additionally, the oxidation of fatty acids [7], degradation of thiamine, cysteine and methionine [8,9], as well as microbial rumen fermentation, are all major mechanisms of flavor generation. Without a doubt, an improved understanding of these flavor-contributing chemical species and processes would provide valuable guidance for developing meat products with a better quality and taste.

Metabolomics is the systematic investigation of all metabolites and their physiological roles in a given biological system. In particular, metabolic profiling has demonstrated a significant utility in helping researchers identify flavor compounds in food and beverage products. For instance, a recent metabolomics study on three different types of white tea, including Silver Needle, White Peony and Shou Mei, revealed broad differences in the levels of amino acids, catechins, nucleosides, phenolic acids and other aroma precursors [10]. Similarly, an investigation of flavor compounds in different fish species by gas chromatography-mass spectrometry (GC-MS) suggested that phosphoric acid, creatinine and lactic acid could play important roles in taste modification [11]. Compared to the conventional sensory assessment methods, which are often subjective and qualitative, metabolomics enables the systematic, objective and quantitative measurement of various sensory properties.

Goat meat, also called chevon, is considered a delicacy in many parts of the world and accounts for around 6% of the global consumption of red meat [12]. Similar to lamb, cooked goat meat produces a complex flavor that is unique from other types of red meat. While this flavor certainly serves as the gastronomic basis for the broad appeal of goat meat worldwide, it could be considered gamey or even disgusting by the unaccustomed. As a result, there has been a continuing effort to understand the various factors that contribute to or alter the flavor of goat meat [1,13]. In the current study, we profiled the metabolites in meat derived from Lubei white goats, Boer goats and Jining grey goats by untargeted liquid chromatography-mass spectrometry (LC-MS). Based on our experimental data, we identified a broad panel of differential metabolites, which included many fatty acids, aldehydes, ketones, lactones, alkaloids, flavonoids, phenolics and drug residues, between the meat specimens of the three goat breeds. The results of our current study provided clear evidence that the metabolic profile of goat meat varies significantly among different breeds, which could be responsible for their distinct tastes and aromas.

## 2. Methods

### 2.1. Animals

All animal experiments were approved by the Animal Care and Use Ethics Committee of the Institute of Agro-food Science and Technology at Shandong Academy of Agricultural Sciences (Projectidentification code:201704-006). The animals evaluated in this study consisted of five Lubei white goats (LBB; Shandong Fengxiang Livestock and Seeds, Laiwu, China), six Boer goats (BE; Shandong Fengxiang Livestock and Seeds, Laiwu, China) and six Jining grey goats (JNQ; Kelong Livestock, Jining, China), all aged less than ten days and housed in standard pens. All goats were fed with the same diet, as described in Table 1.

The meat samples were obtained via a minimally invasive, painless procedure once the goats reached 6 months of age. In brief, each goat was subjected to local anesthesia by injecting 2% lidocaine at a dose of 15 mg per kg of body weight into its latissimus dorsi muscle between the 6th and 10th rib. A semi-automatic biopsy needle (PRECISA, Aprilia, Italy) was then used to resect ~0.1 g of muscle tissues from the anesthetized site. The meat samples were immediately used for metabolite extraction as described below.

### 2.2. Sample Pre-Treatment

For each preparation, 0.08 g of goat meat was weighed and mixed with 1 mL of extraction buffer, which consisted of 70% methanol and 0.1% formic acid in water, in a 2-mL microcentrifuge tube. The sample mixture was vortexed for 10 sec, sonicated for 10 min and then frozen at −20 °C for 1 h. The tube was then thawed to room temperature and centrifuged at 10,000 rpm for 10 min. The supernatant was collected by passing the mixture through a sterile 0.22-μm Millex-GP syringe filter (Millipore, Billerica, MA, USA) and stored at −80 °C.

### 2.3. Liquid Chromatography (LC)

LC was performed on an ACQUITY UPLC I-Class Plus System (Waters, Manchester, UK). Roughly 2 μL of the filtered supernatant described above was directly loaded into an ACQUITY UPLC BEH C18 Column (130 A, 1.7 µm, 2.1 mm × 50 mm; Waters, Manchester, UK) pre-warmed to 35 °C. Separation was performed with a mobile phase consisting of buffer A (0.1% formic acid in water) and buffer B (0.1 formic acid in acetonitrile), at a constant flow rate of 0.4 mL/min. The gradient of B is as follows: 1% at 0 min, 1% to 30% over 1 min, 30% to 60% over 5 min, 60% to 99% over 2 min, and finally 99% to 1% over 2 min.

### 2.4. Mass Spectrometry

Following the LC separation, the metabolites were immediately analyzed on a connected Xevo G2-XS QTof Mass Spectrometer (Waters, Manchester, UK). Full MSe scans were performed using both positive and negative electrospray ionization. The settings used in the MS analysis were as follows: capillary voltage, 3 kV; cone voltage, 30 V; collision energy, 15–45 V; ion source temperature, 120 °C; desolvation temperature, 500 °C; flow rate of cone gas, 50 L/h; flow rate of desolvation gas, 500 L/h; and scan time, 0.2 s. Leu-enkephalin, with a molecular mass of 556.2771 (under the positive-ion mode) and 554.2615 (under the negative-ion mode), was used as an internal MS standard at a concentration of 0.2 μg/mL and a flow rate of 2 μL/min. To ensure data quality for metabolic profiling, six pooled quality control (QC) samples (prepared by mixing all samples) were run. One QC sample was analyzed after every three injections [14].

### 2.5. Statistical Analysis

The raw MS data were first imported into Proteowizard (version 3.0) [15] and converted to the mzXML format. Then, the mass peaks were identified, filtered and aligned by using the XCMS package in R software (version 3.5.0) [16], which yielded data matrices containing the mass-to-charge (*m*/*z*) ratio, retention time and integrated peak intensity of each MS feature. The chemical identity of each metabolite was determined by comparing its m/z value and MS/MS fragmentation pattern to those deposited in the Human Metabolome Database (http://hmdb.ca). The relative level of each metabolite was represented by the intensity of its ion peak. To facilitate the data analysis and comparison, the peak intensities were normalized by using the RFmarkerDetector package (version 1.0.1) [17] in R software based on the method of the z-score [18] and were exported for analysis. In order to capture the global differences between the metabolic profiles of the three goat groups, a principle component analysis (PCA), partial least squares-discriminant analysis (PLS-DA) and orthogonal PLS-DA (OPLS-DA) were conducted with the ropls package (version 1.14.1) in R software [19]. PCA is a dimensionality reduction method based on orthogonal transformation that is often used to visualize relatedness between groups [20]. PLS-DA is a linear classification model that rotates PCA components to obtain a maximum separation between classes. The results of PLS-DA can reveal which variables contain the class-separating information [21]. OPLS-DA is a variant of PLS-DA with distinct predictive and orthogonal components that describe between-class and within-class variance [22]. The fold-change, variable importance in the projection (VIP) score and *p*-value of each metabolite was calculated based on the OPLS-DA model using the ropls package (version 1.14.1) in R software. Differential metabolites were selected according to a *p*-value ≤ 0.05, VIP ≥ 1, and fold change ≥ 2 or ≤ 0.5. The volcano plots used to show these differential metabolites between each two groups were drawn by the ggplot2 package (version 3.2.1) in R software.

### 2.6. Sensory Test

Sensory tests were conducted according to a previously described method [23] with minor modifications. The goat meat samples were rinsed with cold water and cut into 3 cm-long, 3 cm-wide and 2 cm-thick slices, which were then individually and separately placed in boiling water for 90 min. The cooked meat slices were subsequently allowed to cool down, but were used for sensory tests when they were still warm. Ten panelists were trained on the assessment procedure two days prior to the evaluation. During the tests, each participant was required to masticate on at least one sample randomly selected from each experiment group and score it for each taste attribute, including sweetness, bitterness, umami and rancidness/gaminess. The panelists were required to rinse their mouths thoroughly with water after scoring each sample to minimize flavor carryover. The level of each taste attribute mentioned above was scored on a scale of one to ten, with one being the mildest and ten the strongest [23].

## 3. Results

### 3.1. Metabolic Profiling of the Goat Samples

**Figure 1:**
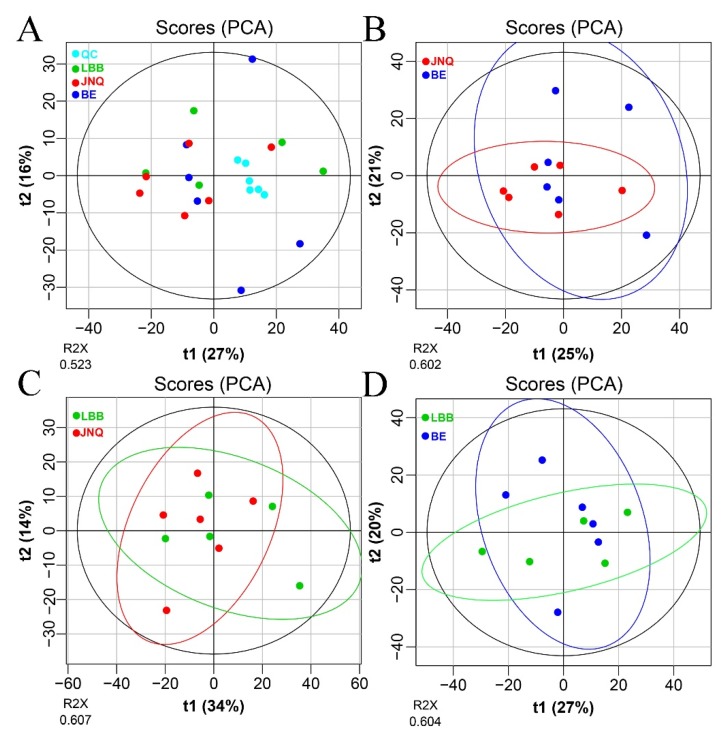
PCA plots showing the different metabolic patterns of goat meat. (**A**) all goat meat samples, (**B**) meat samples from the JNQ group (red) and BE group (blue), (**C**) meat samples from the JNQ group and LBB group (green), (**D**) meat samples from the LBB group and BE group. All with 95% confidence ellipsoids.

**Figure 2:**
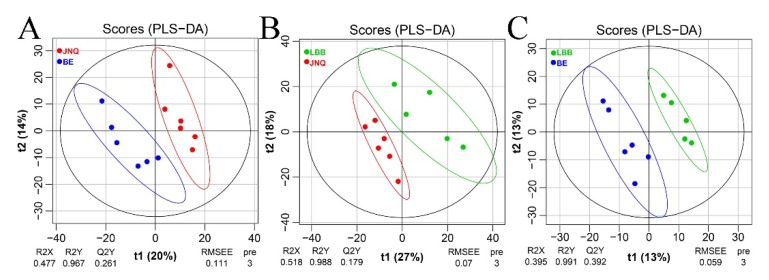
PLS-DA plots showing clear metabolic differences between meat samples The differences between the JNQ group (red) and BE group (blue) was (**A**), (**B**) **-** the JNQ group and LBB group (green), and **C** **-** the LBB group and BE group. All with 95% confidence ellipsoids.

**Figure 3:**
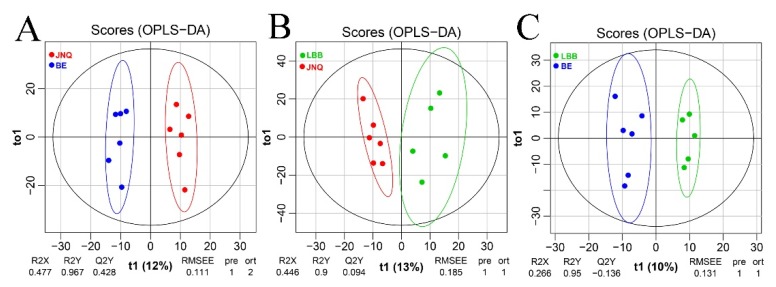
OPLS-DA plots showing clear metabolic differences between the meat samples. (**A**) the JNQ group (red) and BE group (blue), (**B**) the JNQ group and LBB group (green), and (**C**) the LBB group and BE group. All with 95% confidence ellipsoids.

Our untargeted LC-MS approach led to the identification of 977 metabolites out of all 17 goat samples (Table S1). To evaluate the quality of our metabolomics data, we first calculated the coefficient of variation (CV) by mixing all samples together and repeatedly measuring the metabolic profile of the mixture under the standard LC-MS conditions. The obtained CV values were found to be in the range of 1.52% to 8.95%, which confirmed that the measurements were sufficiently reproducible (Figure 1). We then performed a PCA analysis to determine whether there are any global differences between the metabolic profiles of the three goat groups. As illustrated in Figure 1, the three types of goat meat largely overlapped with each other in the PCA plots. A further comparison of each two experiment groups indicated that JNQ and LBB were more similar with each other in metabolic patterns than they were with JNQ (Figure 1). The R2X and Q2 of the different PCA plots were in the ranges of 0.602–0.607 and 0.199–0.300, respectively, suggesting that these models had a satisfactory reliability and predictive ability. A further examination based on the PLS-DA score plots showed a clear separation between each of the two out of the three goat groups (Figure 2). The R2X and Q2 values of the PLS-DA models were in the ranges of 0.602~0.607 and 0.199~0.300, respectively. The OPLS-DA plots also showed a similar trend (Figure 3)

### 3.2. Identification of Differential Metabolites

**Figure 4:**
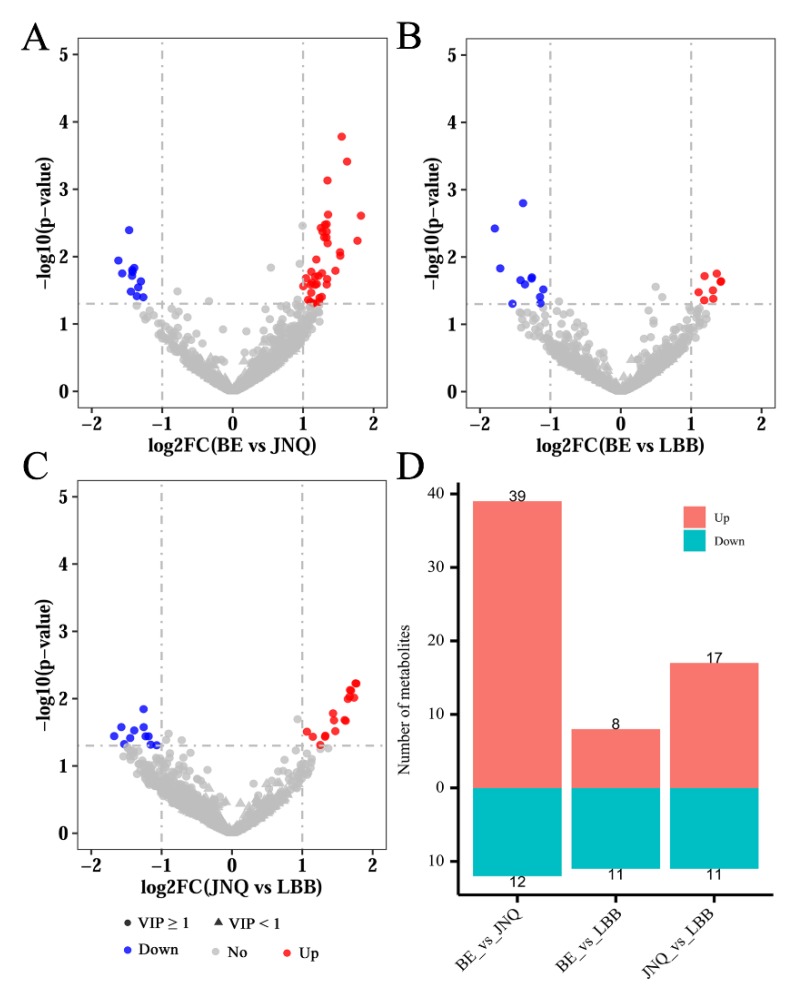
Volcano plots showing the distribution of all metabolites based on their fold-change values (x-axis, on a logarithmic scale), *p*-values (y-axis, on a logarithmic scale), and VIP scores (shape; circle for VIP ≥ 1 and triangle for VIP < 1). A comparison is made between (**A**) the BE group and JNQ group, (**B**) the BE group and LBB group, and (**C**) the JNQ group and LBB group. Up-regulated, down-regulated and non-differential metabolites are colored in red, blue and gray, respectively. (**D**) The numbers of up-regulated (red) and down-regulated (blue) metabolites between each two experimental groups.

**Figure 5:**
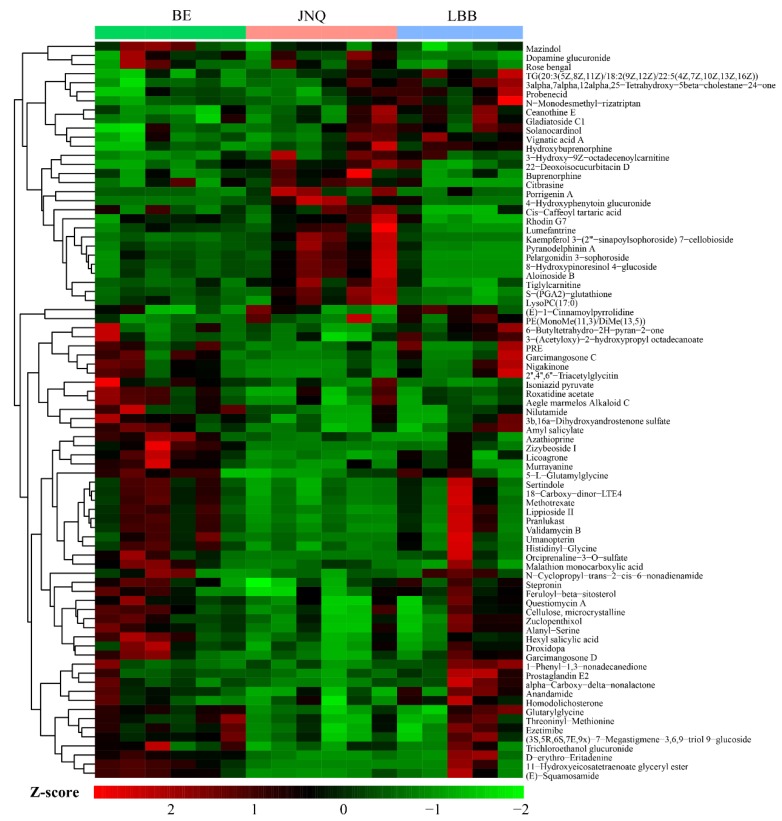
A clustered heat map based on all differential metabolites, which shows clear metabolic differences between the LBB group, JNQ group and BE group. The name of each differential metabolite is provided in the list to the right of the heat map.

Next, we calculated the VIP scores and p values of all detected metabolites based on the OPLS-DA data, as summarized in a volcano plot (Figure 4). Based on the combined selection criteria of the *p*-value ≤ 0.05, VIP ≥ 1, and fold change ≥ 2 or ≤ 0.5, we identified a panel of metabolic features that showed significant differences between at least two of the three experiment groups. A hierarchical clustering analysis also indicated that each type of goat exhibited a distinct metabolic pattern (Figure 5).

### 3.3. Sensory Evaluation

We performed a sensory test on all goat meat samples to further probe the link between their metabolic profiles and taste attributes. As summarized in Table 2, the meat from the JNQ group was considered to be significantly more savory than the samples from the other two groups. The BE group was found to taste more gamey and sweet than the other two, whereas the samples from the LBB group exhibited an increased level of bitterness.

## 4. Discussion

We first surveyed all the differential metabolites and identified a list of compounds with known sensory attributes and/or application in food as flavor modifiers. Compared to that of BEs, goat meat from JNQs contained significantly higher levels of amyl salicylate and hexyl salicylic acid, both of which have been described as “floral”, and lippioside II, a bitter-flavored iridoid. Meanwhile, JNQ-derived goat meat exhibited a lower concentration of deoxoisocucurbitacin D, which is likely to be a metabolite of cucurbitacin, a strongly bitter taste deterrent that plants release in response to animal foraging [24]. Another plant-derived, bitter insect repellent, vignatic acid A, was found to be more abundant in LBBs than in BEs. N-cyclopropyl-trans-2-cis-6-nonadienamide, a food additive with a meaty flavor, was moderately enriched in the meat of LBBs compared to that of JNQs but showed no significant difference between the BE group and LBB or JNQ group. Interestingly, our profiling results also indicated that anandamide was more abundant in BEs and LBBs than in JNQs, whereas the level of dopamine glucuronide was significantly diminished in the LBB group compared to the other two groups. Both anandamide and dopamine function as flavor enhancers rather than serving as flavor constituents themselves. Anandamide can be produced endogenously in the brain as a mood-boosting neurotransmitter, and has been shown to enhance the sensory perception of food and appetite in animals [25]. Anandamide has also been hypothesized to underpin the bliss-inducing property of truffle and dark chocolate [26]. A similar role of dopamine in amplifying positive sensory experiences is also well established [27].

Lipids and fatty acids are some of the main constituents and flavor contributors in meat [1,28]. The metabolomic data in our current study revealed a panel of lipids, fatty acids and their metabolic derivatives that exhibited differential levels between at least two of the three goat groups. Specifically, the LBB meat contained significantly more TG (20:3 (5Z,8Z,11Z)/18:2 (9Z,12Z)/22:5 (4Z,7Z,10Z,13Z,16Z)) and PE (MonoMe (11,3)/DiMe (13,5)), but a decreased level of lysoPC(17:0), compared to that of the BEs. Therefore, the LBBs seemed to show an enrichment of polyunsaturated fatty acids and derivatives, as well as lower concentrations of saturated long-chain carboxylates, in their muscle tissues compared to the BEs. On the other hand, several acylcarnitines, including 3-hydroxy-9Z-octadecenoylcarnitine and tiglylcarnitine, were significantly more enriched in JNQ- than BE-derived goat meat. A comparison between the JNQ group and LBB group indicated that lysoPC(17:0) and tiglylcarnitine were more abundant, whereas the level of 3-acetyloxy-2-hydroxypropyl octadecanoate, a saturated diacylglycerol, declined, in the former group. Previous studies have suggested a link between the lipid composition in meat and the choice of forage or animal feed [29]. For example, Elmore et al. has found that steers fed with cereal-based concentrates exhibited greater levels of α-linolenic acid and derivatives in their muscle tissues compared to those on a diet of grass silage, whereas the concentrations of 1-phytene and n-6 polyunsaturated acids showed a significant increase in the latter group [30]. Meanwhile, Hwang and colleagues reported that grain feeding contributed to elevated levels of monosaturated fatty acids such as oleic acid, but diminished contents of lower saturated fatty acids, in beef compared to foraging [31]. Importantly, the authors suggested a positive effect of monounsaturated fatty acids, especially oleic acid, on the meat flavor, but negative impacts of polyunsaturated and saturated fatty acids [31]. Conversely, linolenic acid has been associated with an undesirable sensory attribute that is often described as “grassy” or “fishy” [32]. On the other hand, breed-specific differences in the fat composition of meat have also been noted [33,34]. Such distinctions have been hypothetically attributed to discrepancies in the expression levels of enzymes involved in the lipid metabolism [35]. Another important mechanism through which fat affects the meat flavor is fatty acid oxidation, which frequently occurs during cooking. Oxidized arachidonic and linoleic acids have been found to produce 2-nonenal, 2,4-decadienal and other carbonyl compounds, which are perceived as “meaty” or rancid in sensory tests [36]. In general, unsaturated fats, particularly polyunsaturated fatty acids, are susceptible to thermal oxidation [37]. Taken together, the overall fat content and the average degree of saturation of the lipid profile are considered as critical factors in governing the flavor differences between the three types of goat meat in our study.

In addition to lipids and fatty acids, volatile carbonyl compounds, including ketones and lactones, also play important roles in imparting a unique aroma and taste attributes on meat products. In the current study, we found that the LBB-derived goat meat contained significantly more 6-butyltetrahydro-2H-pyran-2-one, alpha-carboxy-delta-nonalactone and 1-phenyl-1, 3-nonadecanedione than that from JNQs. In comparison, no noticeable differences in the abundances of these carbonyl compounds were observed between both breeds and BEs. The carbonyl compounds are generally produced from two types of chemical processes, including the Strecker reaction of amino acids in the presence of dicarbonyl compounds (a part of the Maillard reaction), and the oxidation of fatty acids [38]. As already mentioned earlier, aldehydes and ketones produced from lipid oxidation are often the main contributors to increased rancidity in meat, as many of them tend to produce strong, unpleasant flavors. For instance, Kerler and coworkers reported that the increase of trans-4,5-epoxy-(E)-2-decenal and hexanal caused by rancidification played a key role in the quality deterioration of reheated beef [39]. Of course, sensory perception varies considerably among different aldehyde and ketone species. 3-Methyl butanal is a well-studied flavor compound that is commonly described as “creamy”, “nutty” or “tallowy” in taste evaluation tests. It has been discovered that this aldehyde, which can be produced fermentatively by bacteria [40] or non-enzymatically from a Maillard reaction [41], acts as a key contributor to the enhanced flavor of cured meat. Lactones, which are generally formed by the intramolecular esterification of oxidized carboxylic acids, are particularly important flavor molecules due to their low sensory thresholds and have thus been extensively characterized in different food systems [42,43]. Depending on their chemical structures, these cyclic esters confer a wide spectrum of aromas resembling those of coconut (delta-nonalactone and gamma-octalactone), peach (gamma-decalactone), cream (gamma-hexalactone), etc. Matsuishi et al. has demonstrated experimentally that the unusually high levels of coconut- or peach-flavored lactones in Wagyu beef were at least partially responsible for its sweet, appealing aroma [44]. Similarly, 4,5-dihydro-5-propyl-2(3H)-furanone and 4,5-dihydro-5-butyl-2(3H)-furanone have been identified as enhancing the favorable aroma of shallow-fried beef [45]. Combined, the differential profiles of non-lipid carbonyl compounds between the muscle tissues of JNQs and those of LBBs could create subtle nuances in their flavors.

Alkaloids are a large, chemically diverse group of nitrogen-containing compounds that usually confer a bitter taste when ingested [46]. Alkaloids in ruminant meat can be endogenous, but are more often carried over from plant-based animal feed. We identified a large number of alkaloid compounds that exhibited different levels between at least two experiment groups in our study. JNQ-derived goat meat was found to contain lower quantities of alkaloids, including (E)-squamosamide, murrayanine and nigakinone, than those of BEs. On the other hand, the levels of (E)-1-cinnamoylpyrrolidine, vignatic acid A, ceanothine E and solanocardinol were elevated, while those of Aegle marmelos alkaloid C decreased, in the muscle tissues of LBBs compared to those of BEs. In general, there is a negative correlation between the alkaloid content and meat palatability. As an example, Gorecka et al. confirmed that the incorporation of up to 10% of alkaloid-rich lupin hull into minced meat showed no obvious detrimental effect on the latter’s taste; however, increasing the percentage of lupin hull beyond 15% resulted in a sensory panel rejection [47]. Nevertheless, breast and thigh meat from broiler chickens on a standard diet with 30 mg/kg sanguinarine displayed no alkaloid-induced undesirable flavor characteristics [48]. Understandably, the sensory effects of alkaloids on meat would vary significantly according to their chemical nature and doses.

Antioxidants generally do not function as flavoring agents themselves, but instead help preserve the taste of meat mainly by preventing the oxidation of lipids and other chemically labile sensory precursors [49]. Antioxidants in meat are often derived partly from the plant materials in animal feed and partly from additives. We identified at least eight antioxidants, including four phenolics (garcimangosone C and D, cis-caffeoyl tartaric acid, and pelargonidin 3-sophoroside), three flavonoids (licoagrone, pyranodelphinin A and kaempferol 3-(2’-sinapoylsophoroside)-7-cellobioside) and one furofuran (8-hydroxypinoresinol 4-glucoside), among the differential metabolites in this study. On average, the data indicated that the JNQ group had significantly greater levels of cis-caffeoyl tartaric acid, pelargonidin 3-sophoroside, 8-hydroxypinoresinol 4-glucoside, pyranodelphinin A and kaempferol 3-(2’-sinapoylsophoroside)-7-cellobioside, but less garcimangosone D, than the LBB group. The comparison between the BE group and the JNQ group suggested a similar trend, with the exceptions that the concentration of cis-caffeoyl tartaric acid showed no statistically significant difference, and that garcimangosone C and licoagrone were more enriched in the former group. The beneficial effects of antioxidants on the sensory quality of meat are well documented [50]. Thus, the variations in antioxidant levels between different experimental groups in our study could result in different flavor characteristics by affecting the composition of lipids and other carbonyl compounds. It should also be pointed out that many phenolic acids and flavonoids are themselves bitter or astringent, which could further alter the sensory attributes of the meat products.

The metabolomic data confirmed the presence of a variety of drug residues in different goat meat samples that we analyzed in this study. The contamination of animal meat with excessive growth-promoting antibiotics, hormones, potentially toxic pesticides and human pharmaceuticals has become a major food safety concern [51]. Additionally, studies have provided some evidence that even the presence of trace amounts of certain drug residues, such as pesticides, could exert a significant impact on sensory perception [30,52]. Unfortunately, the enrichment of drug residues in animal meat is often associated with anthropogenic rather than natural or innately physiological factors, which renders a systematic evaluation difficult. Though we did not observe any noticeable breed-specific patterns of drug contamination in our study, it is possible for such discrepancies to exist due to metabolic variations of different animal species [53,54].

Our sensory tests, though somewhat subjective, indicated that the three groups of goat meat showed clearly different taste attributes. We then attempted to correlate the metabolic profiles of the meat samples to their sensory properties. To this end, we focused on the top 30 most abundant differential metabolites. As summarized in (Table 3.), Pyranodelphinin A and LysoPC(17:0), the top two most abundant metabolites, considered to have a pleasant, savory taste, were more enriched in meat from the JNQ group compared to that from the other two groups. This coincided well with the sensory finding that the meat samples from the JNQ group were rated as more umami compared to others. In comparison, the trends of other taste attributes were found to be less correlated to the profiles of the corresponding metabolites. For example, the meat samples from the JNQ group contained significantly higher levels of 8-hydroxypinoresinol 4-glucoside and aloinoside B, two of the five most abundant sweet metabolites, than those of the other groups. The other three sweet metabolites, including methotrexate, histidinyl-glycine (considered bittersweet) and alanyl-serine, were more abundant in the BE group than the JNQ group but not the LBB group. As a result, the profile of sweet metabolites did not display a clearly discernible pattern and was not quite consistent with the sensory assessment results showing the LBB group to be the sweetest. This is not unexpected because: i) the meat samples were cooked by boiling, and therefore some of the water-soluble metabolites might be lost to the broth; ii) the abundance of a metabolite is not the only indicator of the strength of its taste characteristics, and taste sensitivity should also be taken into account; and iii) there is evidence that the impact of breed differences on meat taste, though detectible in many cases, is often quite small [1,32]. Nevertheless, these results provide useful guidance as to what metabolites might be responsible for the inter-breed difference in each type of taste attribute. Further research is needed to investigate the contribution of each of these key metabolites to meat taste in a more objective and quantitative manner. 

In summary, we have systematically compared the metabolic profiles of the latissimus dorsi specimens obtained from three popular goat breeds in China. Our metabolomics data revealed significant inter-breed differences in the concentrations of fatty acids, aldehydes, ketones, lactones, flavonoids, phenolics, alkaloids and drug contaminants, which could all contribute to the overall sensory attributes of the meat samples through different mechanisms. These results could provide a useful guidance for developing flavor-enhancing feed formulae or cooking techniques.

## Figures and Tables

**Table 1:** The nutrient composition of the animal feed used in this study, expressed on an air-dry basis.

Constituent	%	Nutritional Value	-
Peanut hay	58.26	Dry matter/(kg·d^−1^)	0.60
Corn	29.60	Digestive energy/(MJ·d^−1^)	6.29
Bran	4.21	Protein/(g·d^−1^)	64
Soybean meal	2.06	Calcium/(g·d^−1^)	4.6
Cottonseed meal	2.03	Phosphorus/(g·d^−1^)	3.0
Calcium bicarbonate	1.09	Sodium/(g·d^−1^)	3.1
Sodium chloride	0.55	-	-
Sodium bicarbonate	0.37	-	-
5% Premix (trace elements, multivitamins, monensin, ion balancer, growth factor)	1.83	-	-

**Table 2:** Sensory test on all goat meat samples.

-	BE	BE ± SEM	JNQ	JNQ ± SEM	LBB	LBB ± SEM
Odor of mutton	6.5	0.21	4	0.17	4.8	0.11
Fresh fragrance	7.5	0.25	7.9	0.22	6.3	0.13
Sweet taste	5.8	0.14	4.2	0.16	3.7	0.08
Bitterness	1.2	0.03	0.5	0.01	1.5	0.05

**Table 3:** 30 differential metabolites in the three types of goat meat with a correlation between the taste attributes and the sensory properties.

Compound	Taste	Classification	BJ *P* Value	JL *P* Value	BL *P* Value
Pyranodelphinin A	Fruit aroma	antioxidants	0.02 *	0.01 **	0.33
LysoPC(17:0)	Help to form an aromatic odor and silky taste during chewing.	lipids	0.07	0.02 *	0.03 *
Roxatidine acetate	Bitter	other 2	0.09	0.97	0.03 *
Gladiatoside C1	Bitter	antioxidants	0.23	0.49	0.05 *
Stepronin	An unpleasant sense of anaesthesia	other 1	0.04 *	0.03 *	0.96
Cellulose, microcrystalline	Unsavory	other 1	0.05 *	0.42	0.30
18-Carboxy-dinor-LTE4	No taste, related to inflammation	lipids	0.00 **	0.13	0.76
22-Deoxoisocucurbitacin D	Unable to retrieve odor-related literature	ketone	0.00 **	0.05 *	0.69
Sertindole	Extremely mild indole odor is a jasmine flower odor, mild cumin odor, moderate foot odor, and severe fecal odor.	other 1	0.01 **	0.08	0.93
Methotrexate	Sweet	alkaloids	0.00 **	0.11	0.83
Ceanothine E	Bitter	alkaloids	0.17	0.47	0.02 *
8-Hydroxypinoresinol 4-glucoside	Sweet	other 2	0.02 *	0.01 **	0.16
Feruloyl-beta-sitosterol	Aroma	other 1	0.12	0.04 *	0.61
1-Phenyl-1,3-nonadecanedione	Meat aroma	ketone	0.35	0.04 *	0.14
S-(PGA2)-glutathione	Unsavory	lipids	0.03 *	0.01 **	0.06
2’’,4’’,6’’-Triacetylglycitin	Umami	lactone	0.02 *	0.09	0.84
(E)-1-Cinnamoylpyrrolidine	Ammonia flavor	alkaloids	0.54	0.20	0.04 *
Pranlukast	Bitter	other 2	0.00 **	0.12	0.83
Vignatic acid A	A faint fishy smell	other 2	0.26	0.33	0.02 *
(3S,5R,6S,7E,9x)-7-Megastigmene-3,6,9-triol 9-glucoside	Bitter	other 2	0.00 **	0.26	0.57
Trichloroethanol glucuronide	Related to animal odor excretion, urine composition	other 2	0.05 *	0.40	0.35
Garcimangosone D	Unable to retrieve odor-related literature	antioxidants	0.04 *	0.05 *	0.75
Histidinyl-Glycine	Bitter-Sweet	other 2	0.00 **	0.18	0.68
Aegle marmelos Alkaloid C	Supposed to be bitter	alkaloids	0.07	0.94	0.02 *
Alanyl-Serine	Sweet	other 2	0.03 *	0.28	0.58
4-Hydroxyphenytoin glucuronide	Unable to retrieve odor-related literature	other 2	0.02 *	0.04 *	0.41
Pelargonidin 3-sophoroside	Bitter	antioxidants	0.02 *	0.01 **	0.09
Aloinoside B	Aloe Vera Fragrance	antioxidants	0.02 *	0.01 **	0.32
3-Hydroxy-9Z-octadecenoylcarnitine	Fishy but aromatic	lipids	0.04 *	0.38	0.07
Homodolichosterone	Unable to retrieve odor-related literature	ketone	0.32	0.03 *	0.14

Note: 1. * means significant difference (*P* ≤ 0.05); ** means very significant difference (*P* ≤ 0.01); 2. BJ means BE vs. JNQ, JL means JNQ vs. LBB, BL means BE vs. LBB.

## Supplementary Materials

The following are available online at https://www.mdpi.com/2218-1989/9/9/176/s1, Table S1: All differential expressed metabolites compared between the two groups.
